# Oily Long-Term Anti-Icing Coating Based on Hydrophobic Cross-Linking Composite Resin

**DOI:** 10.3390/ma18071558

**Published:** 2025-03-29

**Authors:** Zhen Xiao, Mingyang Du, Peining Li, Jingyu Liu, Xiaoyu Tian, Zhi Cheng, Shouren Wang

**Affiliations:** 1 School of Mechanical Engineering, University of Jinan, Jinan 250022, China; 15038776290@163.com (M.D.); lipeining01@163.com (P.L.);; 2 Shandong Key Laboratory of Surface Engineering and Intelligent Equipment for Key Metal Components, University of Jinan, Jinan 250022, China; 3 Sichuan Key Technology Engineering Research Center for All-Electric Navigable Aircraft, Guanghan 618307, China

**Keywords:** anti-icing, oily slow release, hydrophobic, hybrid resins, durability

## Abstract

In this paper, a new type of passive anti-icing coating, i.e., interfacial lubrication coating, is proposed and investigated. The coating was prepared using the spin-coating or drop-coating method, and by adding hydrophobic and lipophilic modified particles to the hybrid resin to lock up the oil, which can significantly reduce the adhesion between the surface and the ice, thus effectively preventing icing. The study systematically characterized the surface morphology, wettability, anti-icing properties, mechanical properties and durability of the four interfacial lubrication coatings. The results show that the hybrid resin-based coating based on fluorinated ethylene–vinyl ester copolymer (FEVE) and polyurethane (PU) exhibits the best anti-icing performance, with ice adhesion as low as 11 kPa and an extended icing delay time of 779 s. Meanwhile, the coating shows excellent long-term stability with virtually no increase in the ice shear strength after being left on the surface for 6 months. The durability mechanism analysis showed that the adsorption of hydrophobic and lipophilic modified nanoporous SiO_2_ on silicone oil and the structural properties of the coating with a dense surface and porous interior are the key factors for achieving the retardation of silicone oil release and maintain the lubricity. This study provides new ideas for the design of efficient and long-lasting anti-icing coatings.

## 1. Introduction

In low-temperature and high-humidity environments, the phenomena of icing and frosting can easily occur, which adversely affect the normal work of transportation equipment such as aircraft, high-voltage transmission lines, instrumentation and machinery, and can even cause serious damage, leading to significant losses of life and property [1,2,3,4]. At present, the main passive anti-icing measures mainly use superhydrophobic coatings and SLIPS coatings for anti-icing [5,6,7,8,9]. However, superhydrophobic surfaces are prone to losing their anti-icing ability in extremely wet and cold environments, and SLIPS coatings cannot prevent ice because of the rapid loss of lubricant due to their extremely poor mechanical properties [10,11,12,13,14,15]. For this reason, a new type of anti-icing coating, called interfacial lubrication coatings, is replacing these two types of coatings for anti-icing [16,17]. An interfacial lubrication coating is a relatively smooth coating constructed by adding silicone oil and other lubricants to the resin binder, which makes it easy for the liquid to slide on the surface and reduces the adhesion between the surface and the ice to achieve the purpose of anti-icing [18,19,20]. Combining the advantages of the two can not only maintain the hydrophobic “rough” surface of the micro-nanostructure [21,22,23,24], but also allows it to retain supercooled droplets formed through direct contact with the liquid–liquid interface which can greatly reduce the adhesion strength of the ice [13], and at the same time, the introduction of an elastomeric substrate, such as a variety of low-surface-energy polymers, resins, etc., can significantly improve the durability of the coatings; as such, the durability of the coating is improved [18].

In recent years, preliminary studies on interfacial lubrication coatings have been initiated. Chen et al. [25] proposed a poly(methyl methacrylate) (PMMA) and pitted 6061 aluminum alloy as the substrate material to reduce interfacial stability by using the expansion force generated by the phase transition, thus reducing the ice’s adhesion strength. Chen et al. [26] constructed a self-sustaining lubrication layer by modifying a solid substrate with a highly hydrophilic polyacrylic acid (PAA)–dopamine conjugate to achieve an anti-ice coating with very low ice adhesion. Golovin et al. [27] tested four PDMS samples with different crosslinking densities, and after introducing the same interfaces to them individually, it was found that the one with the smallest crosslinking density had the smallest ice shear strength, and then silicone oil and PMHS were added to these PDMS samples, respectively, and both additives were able to effectively reduce the ice adhesion by introducing other molecular chains to enhance the interfacial slip capacity. Unfortunately, most of the existing studies on interfacial lubrication coatings have used a single resin for anti-icing, and there is a lack of research on using hybrid resins as a matrix.

Based on the above studies, we prepared hybrid resin-based interfacial lubrication coatings using the spin coating/drip coating method, and characterized and tested the surface morphology, wettability, anti-icing, mechanical properties, and durability of the coatings using different methods, and explored the preparation process and raw material parameter ratios of the coatings. Among them, the best anti-icing performance was offered by the hybrid resin base composed of FEVE and PU, which had an ice adhesion around 11 KPa, an icing delay time of 779 s, and good high-temperature stability, mechanical stability, and durability. After the coating was in place for 6 months, it was found that its ice shear strength, however, hardly increased, and this lubricating coating showed amazing durability. The mechanism of its good durability was analyzed, and it was concluded that the adsorption of silicone oil by hydrophobic and lipophilic modified nanoporous SiO_2_ and the surface-dense internal porous structure of the coating were the main reasons for the slow release of the silicone oil. Such a coating overcomes the shortcomings of previous traditional anti-icing coatings and is a promising candidate for becoming the optimal choice for anti-icing coatings in continuous explorations in the future.

## 2. Materials and Methods

### 2.1. Materials

Fluorocarbon resin (FEVE), polyurethane (PU), acrylic resin (PAA), the corresponding curing agent, dimethylsiloxane (H201-500), and DuPont Fluorine oil (PL100) were provided by Nanjing Evening Clear Chemical Glass Instrument Co. (Nanjing, China). The polydimethylsiloxane (PDMS) and the corresponding curing agent were purchased from Dow Corning (MI, USA). Silica micro powder was purchased from Shanghai Aladdin Biochemical Technology Co. (Shanghai, China), which was oleophilic and hydrophobic (abbreviated as O-SiO_2_).

### 2.2. Preparation of Interfacial Lubricating Coatings

**Figure 1:**
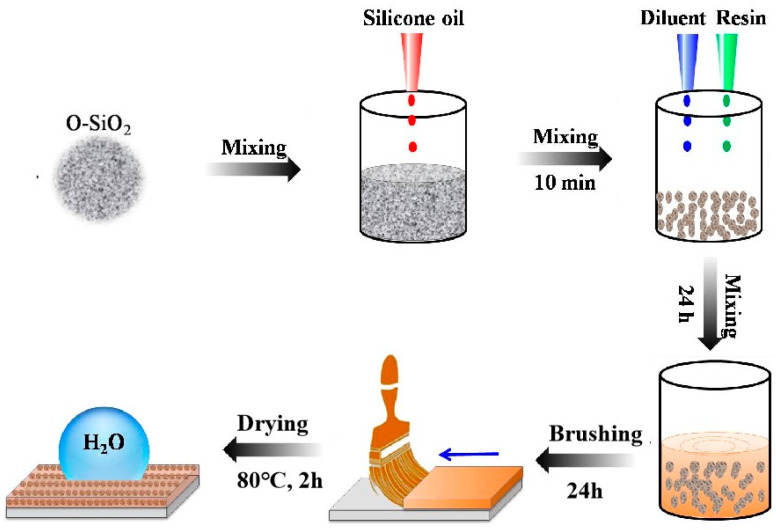
Schematic diagram of the preparation of interfacial lubrication coatings.

The first step was the oil storage treatment of the porous modified powder, which was the key step in the test. First, 2 g of silicone oil was thoroughly mixed with 0.5 g of porous modified powder, and the solution was stirred to make it granular. Then, it was placed in a sealed container, pressurized to 2 MPa, and maintained for 30 min to ensure that the silicone oil fully entered the pores of the granules. Then, 2.5 g of FEVE and 2.5 g of PAA/PU/PDMS were dropped into the beaker with the prepared reservoir particles, and 5 g of FEVE alone was also added into the beaker containing the prepared reservoir particles as a control experiment, along with 5 g of butyl acetate diluent, and the mixtures were stirred well on a magnetic stirrer. Finally, the prepared oil storage particles were added to the diluted resin solution, and a fixed proportion of resin curing agent was added to the mixed solution, which was mixed and stirred well magnetically. The mixed resin-based interfacial lubrication coating was applied to the slide using the drop-coating method, and the coating was flattened and placed in an oven at 80 °C for 2 h to cure, as shown in Figure 1.

### 2.3. Characterization and Testing

The morphology and microstructure of the sample were examined using a field-emission scanning electron microscope (FESEM, FEI, OR, USA). The static contact angle (WCA) and sliding angle (SA) of the droplets on the surface were measured using an OCA 15Pro contact angle meter (DataPhysics Instruments GmbH, Filderstadt, Germany). The average SCA was determined by measuring the same sample at 5 different locations and the average SA was determined by measuring the same sample three times. The significance used for all error bars in this paper is the standard error of the mean. The self-cleaning test was performed by sprinkling a layer of gray powder of varying particle size in the middle of the specimen. A drop of water was dropped onto the top of the specimen with a dropper to observe whether the drop of water could remove all the ash powder in the process of sliding down the specimen and to observe the residual ash powder in the area where the drop of water was sliding down to characterize the self-cleaning ability of the coating. Shore hardness was measured by a Shore hardness tester (LX-D, Shanghai Runyan Machinery Technology Co., Ltd. (Shanghai, China)). Mechanical durability tests were performed with an abrasion meter (BGD 523, Biuged Laboratory Instruments (Guangzhou) Co., Ltd. (Guangzhou, China)) plus a 500 g weight on the composite resin lubricated coating for 100 revolutions. According to GB/T 9286-1998 ‘Scratching Test of Color Paint and Varnish Film’, we adopted the method of cross scratching to test the adhesion of our interfacial lubricating coating. We use a bag cutter to cross-cut the lubricant coating and to try to keep the cutting direction perpendicular to ensure the accuracy of the data. After graphic cutting, we lightly brushed the surface of the coating with a brush, stuck the tape on the surface of the grid, tore off the tape after pressing, and observed the affected area, and according to the completeness of the area, we were able to make a judgment on the adhesion level of the coating.

Ice shear strength was measured by recording the peak value of the force when the ice was detached with a ZTS-50N digital push-pull tester (IMADA, Toyohashi, Japan) in a DC0506 cryostat. The icing–de-icing stability test was performed by recording the value after each de-icing and repeating this cycle 10 times. The delayed icing performance was measured by recording the refrigeration stage using the video contact angle meter, applying a layer of thermal adhesive, and holding the sample in place. A controlled dropper was set to drop 7 μL of water. We set the temperature of the cooling table to −10 °C, started timing when the temperature dropped to 0 °C, and then observed the change in the water droplets, and recorded the time again when the water droplets were completely frozen. The time difference between the two records is the icing delay, and each sample was measured three times, and the average value was taken as the icing delay, which was used to characterize the icing resistance of the samples.

The icing–melting cycle procedure commenced with the uniform application of a heat-conductive adhesive to the base of a Petri dish. Deionized water was subsequently dispensed to fully cover the sample surface, followed by placement of the dish in a refrigeration chamber preset to −18 °C for 2 h. The frozen specimen was then transferred to a forced-air drying oven operating at 80 °C for 30 min to ensure complete ice melt. Upon completing each cycle, samples were retrieved for systematic evaluation of surface wettability and ice-resistant characteristics. This sequential process of cryogenic conditioning, thermal recovery, and performance characterization was iteratively executed across 10 consecutive cycles.

## 3. Results and Discussion

### 3.1. Subsection Surface Layered Structure of the Coating

**Figure 2:**
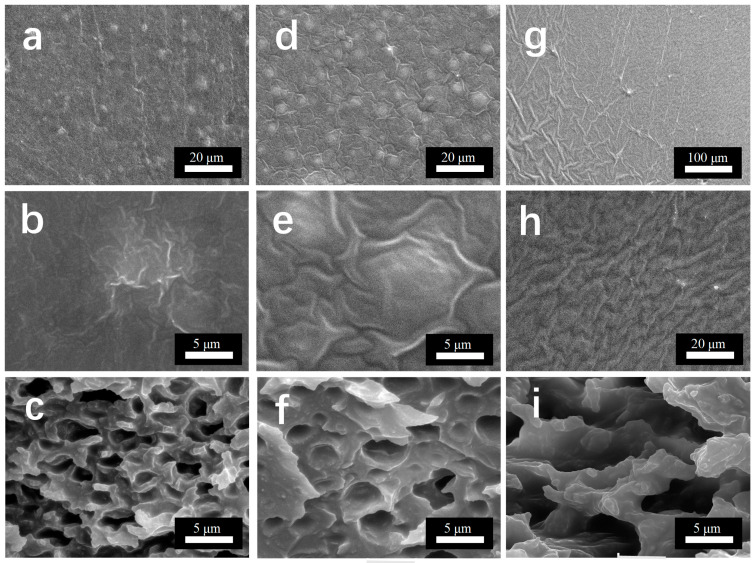
SEM images of hybrid resin-based lubrication coatings. (**a**,**b**) Planar SEM images of FEVE + PAA; (**c**) cross-sectional SEM images of FEVE + PAA; (**d**,**e**) planar SEM images of FEVE + PU; (**f**) cross-sectional SEM images of FEVE + PU; (**g**,**h**) planar SEM images of FEVE + PDMS; (**i**) cross-sectional SEM images of FEVE + PDMS.

The surfaces of three different resin-based interfacial lubrication coatings were morphologically analyzed using a Sirion field emission scanning electron microscope. SEM images at different magnifications were obtained, as shown in Figure 2. It can be seen that the silica micro powder forms certain agglomerates with the hybrid resin, and the agglomerated silica micro powder has a micrometer size, and is scattered on the surface, and the particle size of the agglomerated particles is different, as shown in Figure 2a,d. The hybrid resin shows a better encapsulation of the silica micro powder, and it can be seen that the agglomerated silica micro powder is well encapsulated by the resin base, as in Figure 2b,e. This facilitates the preservation of the surface morphology, and there may be a certain enhancement of its durability and mechanical properties. However, in the FEVE + PDMS coating, there are fewer agglomerates, and a “river” surface morphology is observed, which is presumed to have a thicker layer of silicone oil covering the surface, which may have a certain effect on the mechanical properties. In Figure 2c,f,i, it can be observed that the interior of the coating features a loose and porous structure, which facilitates the release of silicone oil from the reservoir particles.

**Figure 3:**
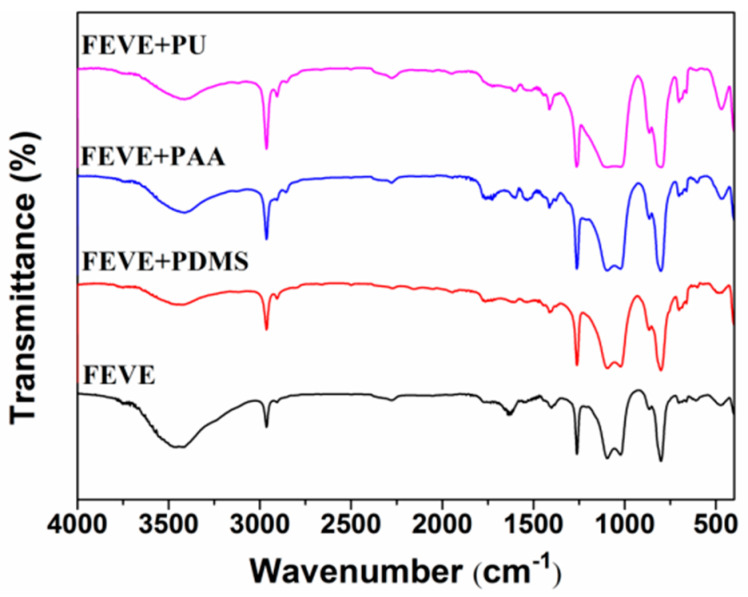
Infrared spectra of four different resin-based coatings.

A comparison of the infrared spectrograms of the fluorocarbon resin base and the other three hybrid resin bases was made. As shown in Figure 3, it was found that the peaks for the four resin-based coatings were very similar, and at an absorption wavelength of around 1370 cm^−1^, the position should be the absorption peak of methyl-CH_3_, which is due to the incorporation of the silicone oil introducing methyl pairs throughout the lubricating coating’s system. At around 3500 cm^−1^ the absorption peak was stronger for fluorocarbon resin-based coatings, possibly -OH or -NH_2_, due to the cross-linking of molecular chains that occurs due to the introduction of different polymer chains, which affects the free state of this functional group. A new Si-CH_3_ peak was added to FEVE + PDMS at around 1260 cm^−1^, which changed the original peak and imparted properties such as low surface energy to the coatings. The C=O carboxylate peak appeared in FEVE + PAA at an absorption wavelength of around 1700 cm^−1^, which affected the hydrophilicity and activity of the coatings.

### 3.2. Surface Wettability and Self-Cleaning of Composite Coatings

**Figure 4:**
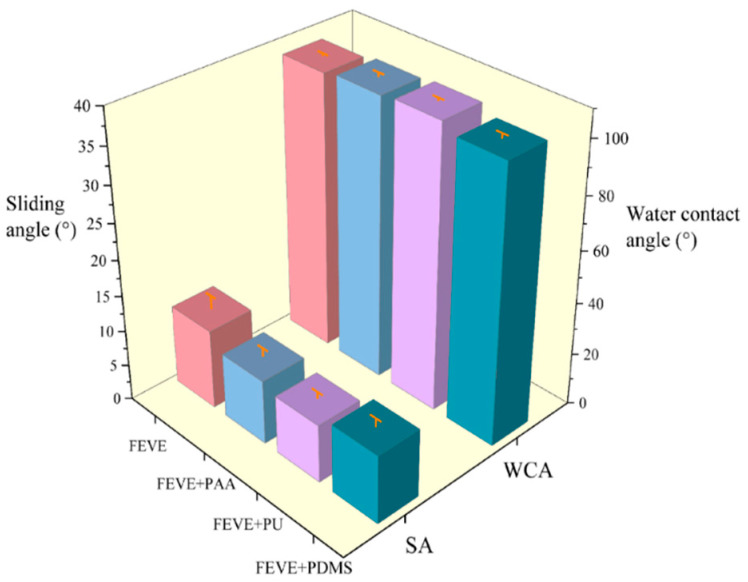
Comparison of contact angle and sliding angle of four different resin-based interfacial lubrication coatings. (The meaning of the error bars is the standard error of the mean).

The static contact angle and sliding angle of the coatings with different resin bases were recorded, as shown in Figure 4. From the figure, it can be seen that FEVE + PU has the best wetting performance with the largest droplet contact angle, the smallest sliding angle, and the best wettability, and all four coatings reached the hydrophobic state, and it was found that the lubrication performance of the coatings becomes better to varying degrees with different resin blends as compared to a single fluorocarbon resin.

**Figure 5:**
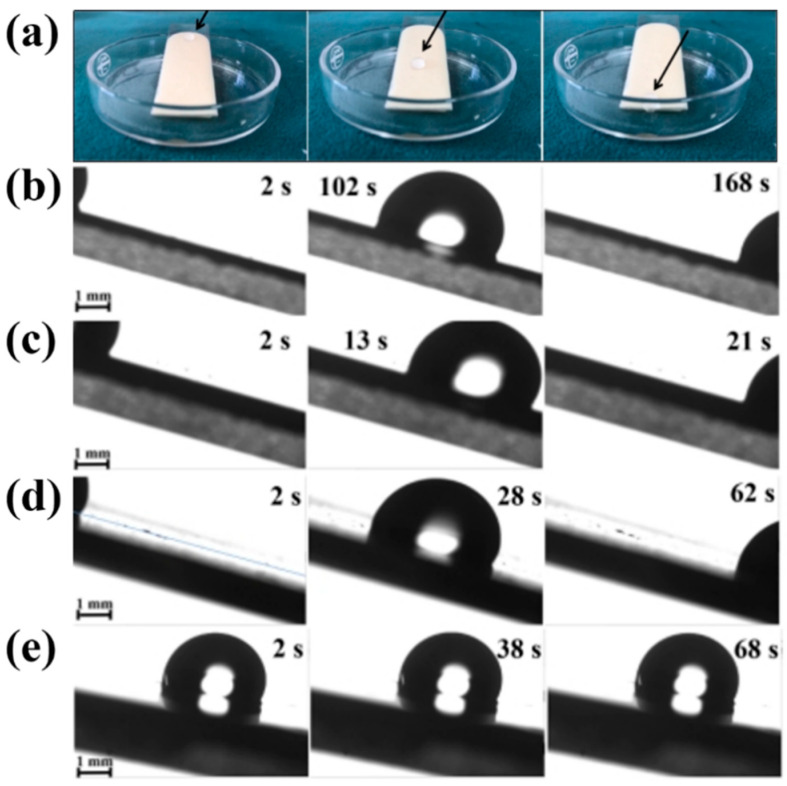
(**a**) Physical image of dynamic wettability characterization of FEVE + PDMS hybrid resin-based lubrication coatings. Optical images of four different resin-based interfacial lubrication coatings with slopes falling (at an angle of about 15°): (**b**) FEVE; (**c**) FEVE + PU; (**d**) FEVE + PAA; (**e**) FEVE + PDMS.

A slope hydrophobicity test was conducted on the FEVE + PDMS sample with the largest sliding angle among the three hybrid resin-based coatings, and the water droplet sliding process was documented with photographs, as seen in Figure 5a, where the water droplets slid down to the bottom in tens of seconds, proving that the hybrid resin-based lubrication coatings all have excellent hydrophobicity. To assess the hydrophobicity more accurately, the water droplet sliding process was recorded using a video contact angle meter, as shown in Figure 5b–e. This recording details the falling time and distance of various lubricant coatings, and can be used to characterize the level of hydrophobicity. From the figure, it can be seen that the lubricating coatings mixed with different resin bases all have better hydrophobicity than the single fluorocarbon resin-based coatings, and the hydrophobicity of FEVE + PU is the best, and the falling speed reaches 0.59 mm/s, which shows an excellent hydrophobicity performance.

**Figure 6:**
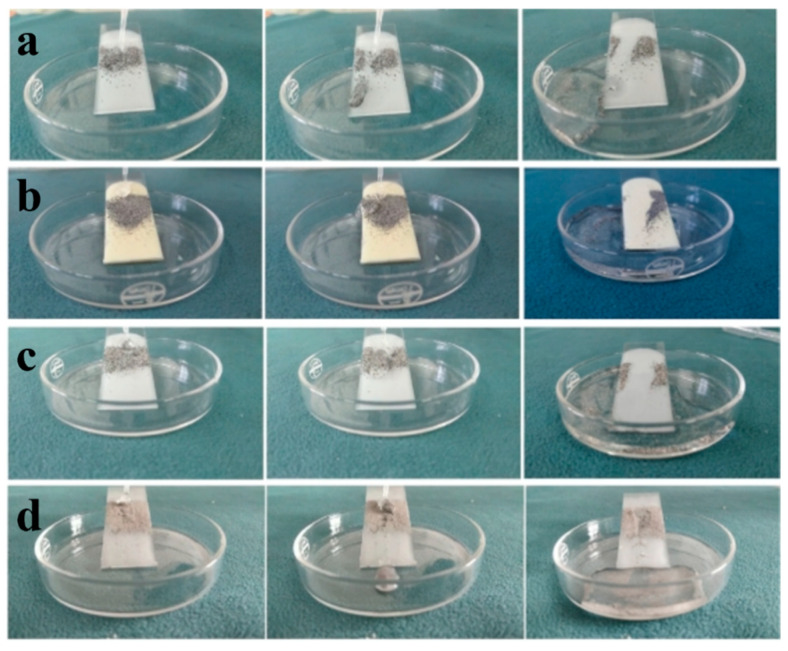
Self-cleaning performance corroboration plots for four coatings: (**a**) FEVE + PDMS; (**b**) FEVE + PU; (**c**) FEVE + PAA; (**d**) FEVE.

The self-cleaning effect of the coatings was evaluated through a simple simulation experiment to examine the self-cleaning ability of different hybrid resin-based coatings, as shown in Figure 5b–e. In this experiment, the falling time and distance of various lubricant coatings were recorded in detail, through which the level of hydrophobicity could be characterized. As shown in Figure 6a–c, water droplets were able to roll off the surfaces of the three interfacial lubrication coatings smoothly. They successfully carried away the dust, leaving no residual ash, which indicates that these coatings possess a self-cleaning performance similar to that of a lotus leaf. In contrast to the single FEVE coating (see Figure 6d), the self-cleaning property of the interfacial lubrication coatings is superior.

### 3.3. Long-Term Anti-Icing Behavior and Mechanisms of Action

**Figure 7:**
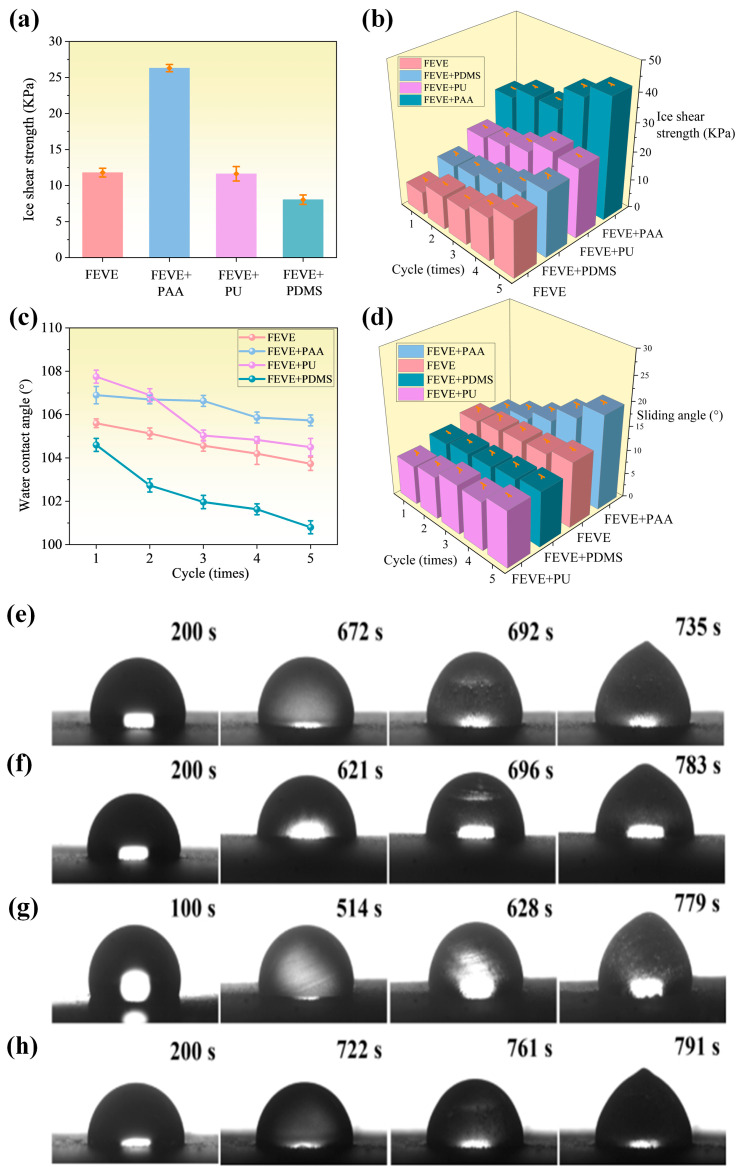
(**a**) FEVE + PDMS, (**b**) FEVE + PU, (**c**) FEVE + PAA, and (**d**) FEVE. (**a**) Comparison histogram of ice shear of four different resin-based interfacial lubrication coatings, (**b**) Images of ice shear of four different resin-based interfacial lubrication coatings with the number of icing–de-icing cycles. Curves of wettability of four different resin-based interfacial lubrication coatings with the number of icing-melting cycles. (**c**) Contact angle. (**d**) Sliding angle optics of icing delay records of four different resin-based interfacial lubrication coatings Images. (**e**) FEVE, (**f**) FEVE + PAA, (**g**) FEVE + PU, and (**h**) FEVE + PDMS. (The meaning of all error bars is the standard error of the mean).

The ice-phobic nature of different hybrid resin-based lubrication coatings was characterized by comparing their ice shear values, as shown in Figure 7a. It can be observed that the ice-phobic nature of the FEVE + PAA hybrid resin-based coatings did not reach a level of greater than 20 KPa. In contrast, the other coatings exhibited smaller ice shear values and demonstrated considerable ice-phobic properties. The hybrid resin bases of FEVE + PU or FEVE + PDMS all had a significant effect on the ice-phobic properties of the materials. However, FEVE + PAA had a decreased ice-removal ability compared to single fluorocarbon resin-based coatings. It is speculated that the polymer chains introduced by PAA increase the cross-linking density of polymer chains in the coatings. This increase leads to a decrease in the mobility of molecular chains on the surface, and consequently, the ice-removal ability deteriorates.

As can be seen in Figure 7b, the ice shear strength of the four samples increased to varying degrees with the increase in the number of icing–de-icing cycles. Specifically, the ice shear strength of the sample with FEVE + PAA began to increase significantly after the third cycle, indicating that the surface structure started to be disrupted, which in turn affected its anti-icing performance. In contrast, the other samples exhibited better icing–de-icing cycle stability within five cycles, and almost all of them could maintain an ice shear strength at around 20 KPa.

From Figure 7c,d, it can be seen that the sliding angle of the four different resin-based lubricant coatings increases with the increase in the number of icing–deicing cycles, and the contact angle becomes smaller and smaller. Among them, the contact angle of the FEVE + PAA sample does not change much, but the sliding angle has increased greatly, and hydrophobic ice-phobic properties have decreased. Combined with the inherently brittle characteristics of the material PAA, we find that the anti-icing ability of the PAA and FEVE hybrid resin indeed does not reach a satisfactory standard. The other two hybrid resins show better icing–melting cycling stability up to five cycles and a better cycling performance than single FEVE, indicating that the introduction of the other two polymer chains increases the ice-surface interfacial mobility and improves the ice-hydrophobicity.

The icing delay of four different resin-based lubrication coatings was characterized and recorded in Figure 7e–h. All four lubrication coatings can prolong the icing time on the surface, and by mixing the resins, the icing delay time of the coatings becomes longer, and the anti-icing ability is improved. Comprehensively, the hybrid resin-based coating of FEVE + PU has the most excellent anti-icing ability, with a small ice shear and a longer icing delay time, which is excellent for anti-icing. On the whole, the anti-icing ability of the FEVE + PU hybrid resin-based coating is the most excellent; its ice shear force is small, and the icing delay time is long, making it an excellent material for anti-icing.

### 3.4. Mechanical Stability

#### 3.4.1. Mechanical Properties

**Figure 8:**
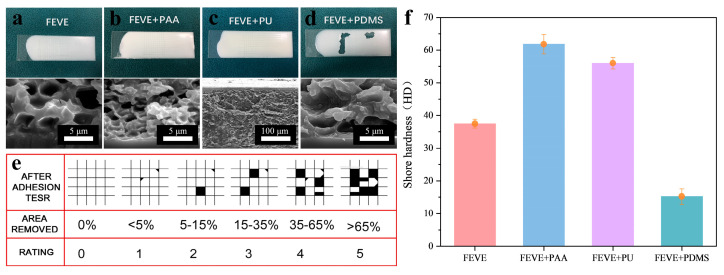
Images of adhesion test and SEM at the bottom of the cross-section for four different resin-based interfacial lubrication coatings: (**a**) FEVE, (**b**) FEVE + PAA, (**c**) FEVE + PU, and (**d**) FEVE + PDMS, (**e**) Corresponding adhesion levels. (**f**) Shore hardness histograms of coatings for four different resin bases. (The meaning of the error bars is the standard error of the mean).

Figure 8a–d are physical drawings of the four different resin-based lubrication coatings at the end of the scribing test, and the coatings were graded according to the corresponding levels of adhesion, as shown in Figure 8e. It can be seen that the adhesion levels of FEVE, FEVE + PAA, and FEVE + PU all reach grade 1, but the one for FEVE + PDMS has very obvious traces of peeling off, and the adhesion level is grade 3–4, which is poorer in adhesion, poorer in utility, and weaker in mechanical properties. Through the sample below for the bottom of the coating, a cross-section of the SEM pictures can be seen, although the middle of the coating is loose and porous, the early surface will form a layer of dense film, and improve and enhance the adhesion of the sample. The formation of this dense film is due to the large surface energy because of the large contact area with the coating, which prioritizes the formation of the film at the bottom. The reason for the low adhesion of the coating of FEVE + PDMS is supposed to be the softness of PDMS, followed by the small polar interaction between this polymer and the slide molecules and less adhesion.

Combined with the Shore hardness of the four resins in Figure 8f, it can be seen that FEVE + PDMS has the lowest hardness, which is due to the softness of PDMS and its similar composition to silicone oil. Combined with the planar SEM image in Figure 2, the same amount of silicone oil is added, but the oil film on FEVE + PDMS is thicker, which weakens the mechanical properties even more.

#### 3.4.2. Durability

**Figure 9:**
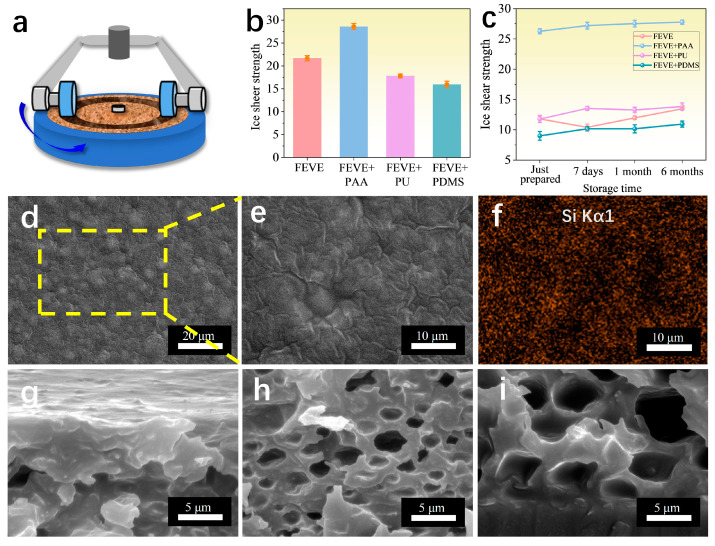
(**a**) Schematic of wear. (**b**) Ice shear strength of four different resin-based lubricated coatings after 100 revolutions of wear with a 500 g weight. (**c**) Dot line plots of ice shear strength of different resin-based interfacial lubricated coatings after being left for different times. (**d**,**e**) Comprehensive analysis of the SEM images of the hybrid resin of FEVE + PU. (**f**) Characterization of Si Kα1 corresponding to EDS spectral scans of the region of Figure 9e. (**g**–**i**) SEM images of the top, middle, and bottom of the coating cross-section. (The meaning of all error bars is the standard error of the mean).

Figure 9a shows a wear diagram; the ice shear of the different resin-based interfacial lubrication coatings before and after wear did not change much compared to Figure 7a, as shown in Figure 9b. Since the microstructure of the surface of the coating is always depleted in different forms when in contact with the external environment, we wanted to test what effect such an external environment would have on the coating and characterize the ice adhesion strength of different resin-based interfacial lubrication coatings after a longer period, as shown in Figure 9c, where the ice shear force was tested on samples that had been freshly prepared, left for 7 days, left for 1 month, and left for 6 months, respectively. It was found that the ice protection ability did not decrease much and remained almost at the same level despite the long period of six months’ placement, except for the FEVE + PAA hybrid resin-based interfacial lubrication coating, which showed a decrease in surface molecular chain mobility due to an increase in the cross-linking density of the polymer chains in the coating due to the introduced polymer chains, the ice shear strength of the other resin-based interfacial lubrication coatings remained below 20 KPa after six months of placement.

In order to analyze the reason for the long durability of the lubricant coatings, a more in-depth study of their microstructures was carried out. As shown in Figure 9a–i, the scanning electron microscopy results of the coating of FEVE + PU can illustrate the durability mechanism of the interfacial lubrication coating. From Figure 9d,f, it can be seen that the resin-coated porous oil-storing silica micro powder is uniformly distributed on the surface, which builds up a low surface energy structure conducive to ice evacuation, and the silica oil forms a thin film distributed on the whole surface of the coating, which enhances the abrasion resistance and reduces the droplet “pinning” point during icing, which improves the ability to prevent over-icing. Moreover, the reason that such an ability can be maintained for a long time lies in the structure of the coating, as can be seen from Figure 9g–i, the structure of the coating is a roughly “dense film—porous oil storage structure—dense film”, with the top and the bottom taking the lead due to the higher surface energy during curing, and the middle portion of the coating is cured first due to the modified hydrophobic and lipophilic porous silica micro powder. The middle part, due to the presence of modified hydrophobic and lipophilic porous silica micro powder, forms a porous structure in synergy with the polymer chains in the resin, which can encapsulate the silicone oil and silica micro powder well. Since the interior and the surface are connected by the silicone micro powder-coated resin, when the silicone oil on the surface is depleted due to the existence of surface tension, the silicone oil stored in the interior will go along the small pores to the surface until the surface and the interior reach a dynamic equilibrium, and at this time, the interfacial lubrication coating has a good ability to prevent ice-covering again. This is the general mechanism behind the amazing durability of interfacial lubrication coatings.

## 4. Conclusions

Hybrid resin-based interfacial lubrication coatings were successfully prepared using the spin/drip coating method, and the performance of the hybrid resin-based lubrication coatings of FEVE + PAA, FEVE + PDMS, and FEVE + PU were tested and characterized through controlled experiments, respectively.

(1) Through comparisons, it was found that the hybrid resin-based lubrication coating of FEVE + PU had the most excellent overall performance. Its static contact angle and sliding angle can reach up to 107.8° and 8° at best, which achieves excellent hydrophobicity.

(2) Most importantly, the ice shear strength can still be maintained below 20 KPa over five icing–melting/de-icing cycles, and the lowest can reach 10.6 KPa, with an excellent anti-icing ability, and the delay time of icing is also up to 791 s. It has excellent high-temperature stability and mechanical stability.

(3) After six months of placement, its anti-icing ability has not been reduced too much, nor has its ice shear strength; in addition to the introduction of polymer chains, the coating of polymer chain crosslinking density increased, resulting in the surface of the molecular chain mobility decreasing in the FEVE + PAA hybrid resin-based interfacial lubrication coatings, and the other hybrid resin-based interfacial lubrication coatings were able to maintain a shear strength below 20 KPa.

The hybrid resin of PU and FEVE showed a strong anti-icing performance throughout the test and this has practical value, but more exploration needs to be carried out as to whether the frost phenomenon and ultra-low temperature will have an effect on its anti-icing performance, mechanical properties and durability in extremely cold and humid environments.

## Data Availability

The original contributions presented in this study are included in the article. Further inquiries can be directed to the corresponding author.
